# A self-octaplexing millimeter-wave antenna array for 5g fr2 spectrum

**DOI:** 10.1038/s41598-025-94786-5

**Published:** 2025-03-26

**Authors:** Gunjan Srivastava, Amit Kumar, Akhilesh Mohan, Sachin Kumar, Tanweer Ali

**Affiliations:** 1 Department of Electronics and Communication Engineering, Graphic Era (Deemed to Be) University, Dehradun, 248002 India; 2 Department of Electronics and Communication Engineering, Indian Institute of Technology Roorkee, Roorkee, 247667 India; 3 Department of Electronics and Communication Engineering, Galgotias College of Engineering and Technology, Greater Noida, 201310 India; 4 Department of Electronics and Communication Engineering, Manipal Institute of Technology, Manipal Academy of Higher Education, Manipal, 576104 India

**Keywords:** Energy science and technology, Engineering

## Abstract

In this article, a quarter mode substrate integrated waveguide (QMSIW) self-octaplexing antenna array for fifth generation (5G) millimeter wave communications is presented. It consists of microstrip fed eight QMSIW antenna arrays to obtain the electromagnetic radiations at eight distinct frequency bands that lies in n257, n258, n259 and n260 of 5G spectrum for self-octaplexing operations. The designed self-octaplexing antenna array utilizes TE_110_ mode of the QMSIW resonator. The designed antenna array radiates at 25.8, 27.5, 29.5, 31, 35.6, 36.8, 38.2 and 39.7 GHz with the corresponding gains of 9.7, 10.7, 10.9, 11.0, 11.4, 11.7, 11.9 and 12.2 dBi, respectively. High inter-port isolations (> 33 dB) are obtained over all the operating bands of the self-multiplexing antenna array.

## Introduction

Multiband antennas are preferred over single band antennas, as they can be utilized for a variety of applications^1–3^. However, they require additional circuit elements such as multiplexers with high isolations for the selection of the particular bands that eventually makes the system bulky, lossy and costly^4,5^. Moreover, the simultaneous transmission and reception of the signals is not feasible in multiband antenna systems. To overcome the aforementioned issues, self-multiplexing antennas are one of the best solutions. Self-multiplexing antennas not only overcome the requirement of external multiplexer but also provide compact solution along with high inter-port isolations. Numerous substrate integrated waveguide (SIW) self-multiplexing antennas are reported at microwave bands in the literature. The reported self-multiplexing antennas operate at two^6–9^, three^10,11^, four^12–18^, five^19^, six^20–23^and eight^24–27^ distinct frequency bands of the microwave spectrum.

Different types of slots are etched on the top surface of the SIW cavity to obtain several self-diplexing antennas, which includes rectangular slots^6^, square ring-shaped slots^7^, Y-shaped slots^8^and U-shaped slots^9^. Self-triplexing antennas are also reported in the literature that utilizes the hexagonal and rectangular SIW cavities^10,11^. Numerous self-quadruplexing antennas are designed and developed in the literature^12–18^. They can be designed with the usage of slots and patches of different dimensions introduced over the SIW cavity. The operating frequencies of self-quadruplexing antennas in references^12–14^are obtained by introducing the T-shaped, V-shaped and L-shaped slots over top surface of the SIW cavities. Apart from slot-based designs, the different operating frequencies can also be attained by varying the width of the SIW cavity backed patches^15^and by utilizing four distinct quarter-mode SIW (QMSIW)^16,17^cavity resonators. A microfluidic frequency reconfigurable self-quadruplexing antenna is designed and developed in reference^18^.

A compact self-quintuplexing antenna for penta-band applications is presented in reference^19^. A π-shaped slot and a T-shaped slot are etched over the rectangular SIW cavity to obtain five distinct frequencies. A few self-hexaplexing SIW antennas are also reported^20–23^, which operate in six distinct frequency bands. In reference^21^, a self-hexaplexing antenna utilizes TE_110_, TE_120_ and TE_210_modes of the rectangular SIW cavities for its operation in six distinct frequencies in S- and C-bands. In reference^22^, self-hexaplexing antenna operation in sub-6 GHz is reported that uses one-sixth mode of the hexagonal cavity. Another SIW based hexaplexing antenna operating is designed in the literature which utilizes four square-like quarter-mode SIW (S-QMSIW) resonators, and two triangular quarter-mode SIW (T-QMSIW) resonators^23^.

Recently, self-octaplexing antennas for octa-band operations are also reported^24–27^. An eighth mode SIW self-multiplexing antenna for sub-6 GHz wireless applications is designed in reference^24^. It has the limitation of having too many design parameters to achieve frequency tunability; specifically, the slot length and slot position must be changed to obtain the desired operating bands. In reference^25^, a SIW-microfluidic frequency-tunable self-octaplexing antenna is designed and developed. The ability to tune each resonant frequency is achieved by using solid dielectrics with varying permittivity into the specific carved region of the antenna. The carved regions are of depth 0.5 mm in the substrate thickness of 0.787 mm, resulting in the complex system design, which requires complex fabrication along with a higher cost. In reference^26^, a compact self-multiplexing antenna for eight sub-6 GHz distinct frequency bands is reported.

Nowadays, the millimeter wave spectrum of fifth generation (5G) wireless communication is being exploited as it offers larger bandwidth, high data rates and large channel capacity^28,29^. Two SIW based self-quadruplexing antennas for two microwave bands and two-millimeter wave bands are designed and developed in references^30,31^. A self-quadruplexing antenna in which all the bands operate only at millimeter wave frequencies is also reported^32^. However, the realized gains of these millimeter wave self-multiplexing antennas are limited that restricts its applications where high gains are required. To overcome the gain limitations at millimeter wave frequencies, antenna arrays are the potential candidate. To the best of authors’ knowledge, self-multiplexing antenna array for millimeter wave applications is not reported so far in the literature.

In this article, a compact self-octaplexing antenna array for 5G n257, n258, n259 and n260 bands is presented. It utilizes TE_110_ mode of QMSIW resonators to radiates at eight distinct frequencies of 5G FR2 bands. The eight distinct frequencies of the antenna array are obtained by altering the QMSIW resonators. High isolations (> 33 dB) are accomplished among the different ports of the designed antenna array. Some of the key contributions of the proposed work are as follows:

- The proposed antenna design provides self-multiplexing operations at eight distinct frequencies (25.8, 27.5, 29.5, 31, 35.6, 36.8, 38.2 and 39.7 GHz) for millimeter wave applications.
- Each band of the proposed antenna can be independently tuned within 5G FR2 spectrum.
- The designed antenna possesses excellent gains (9.7, 10.7, 10.9, 11.0, 11.4, 11.7, 11.9 and 12.2 dBi) in eight distinct operating bands of self-multiplexing antenna.
- The designed self-multiplexing antenna have high inter-port isolations (> 33 dB) without any decoupling structure.

To the best of the authors’ knowledge, this is the first instance when the self-octaplexing antenna array for 5G FR2 bands is designed and developed.

## Antenna configuration

**Fig. 1:**
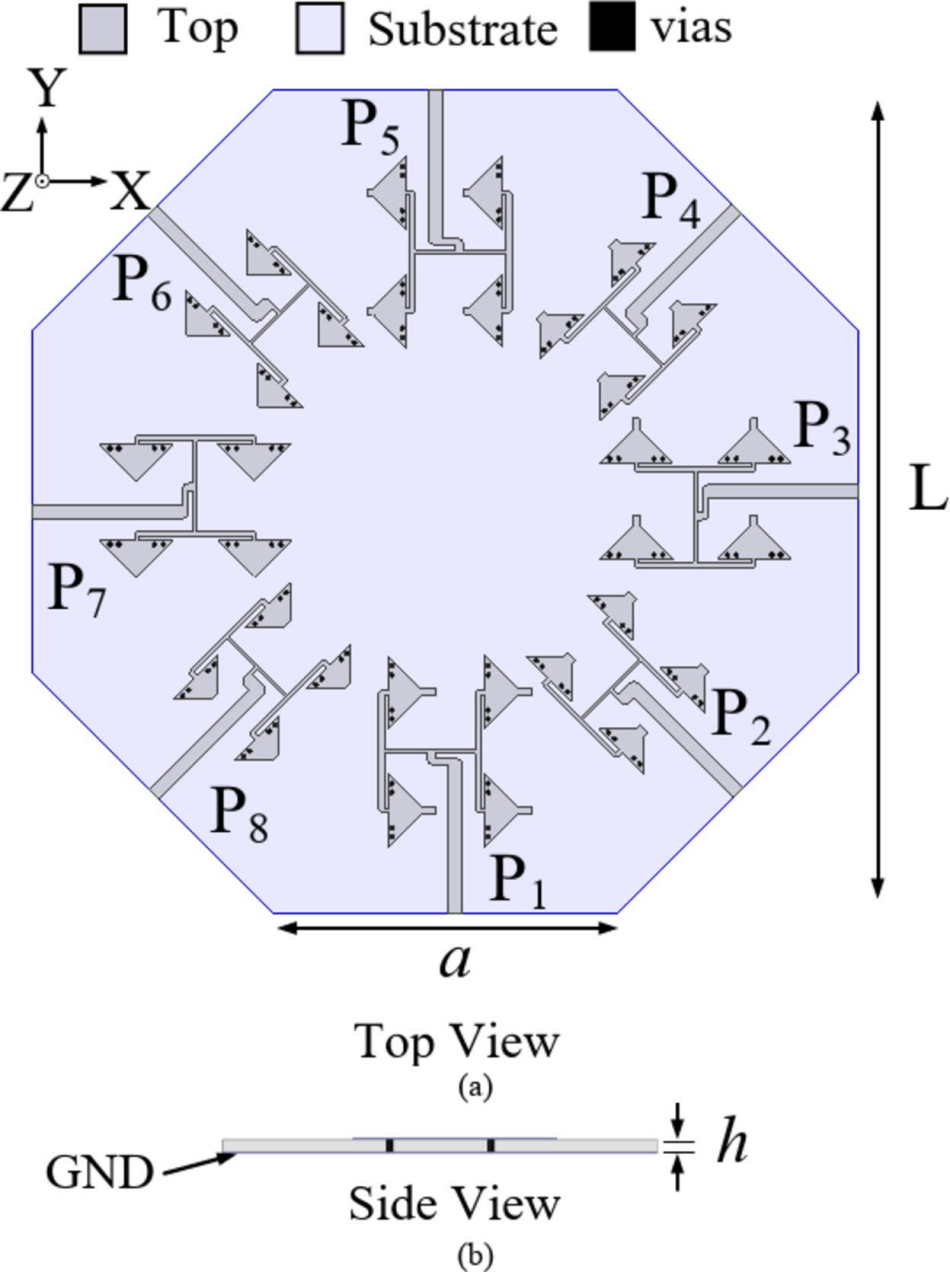
Proposed Self-octaplexing antenna array: (**a**) Top view, (**b**) Side view (*L* = 46 mm, *a* = 19 mm, *h* = 0.254 mm, vias diameter = 0.25 mm, vias separation = 0.5 mm).

Figure 1 shows the layout of the proposed self-octaplexing millimeter wave antenna array for 5G FR2 bands. It is designed on 0.254 mm thick Rogers RT/duroid 5880 substrate (*ε*_*r*_ = 2.2 and tan *δ* = 0.0008). The designed antenna array operates in n257, n258, n259 and n260 of 5G FR2 bands. The designed self-octaplexing antenna array consists of eight distinct 2 × 2 QMSIW antenna arrays. The distinct 2 × 2 QMSIW antenna arrays are placed across the edge *a* of the regular octagon and is fed through a 2 × 2 corporate feed network. The TE_110_ mode of the QMSIW resonator is utilized in the present work.

### Operational mechanism

In this section, the operational mechanism and design methodology of the proposed self-octaplexing antenna array for 5G FR2 bands is presented in detail.

## QMSIW cavity resonator

**Fig. 2:**
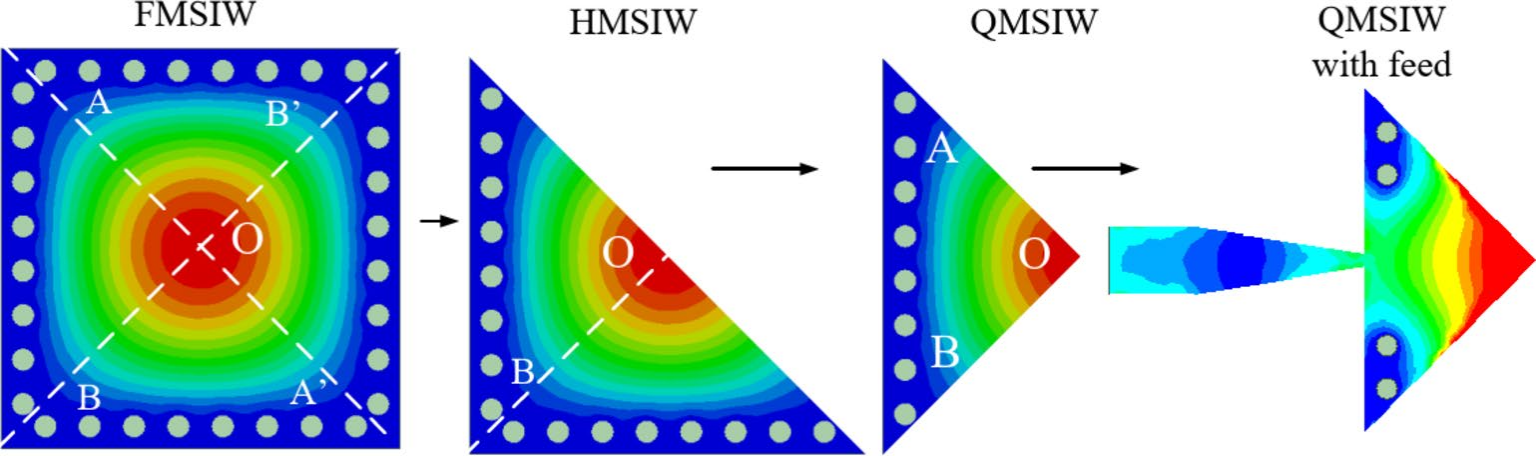
Evolution of QMSIW cavity resonator with feed.

**Fig. 3:**
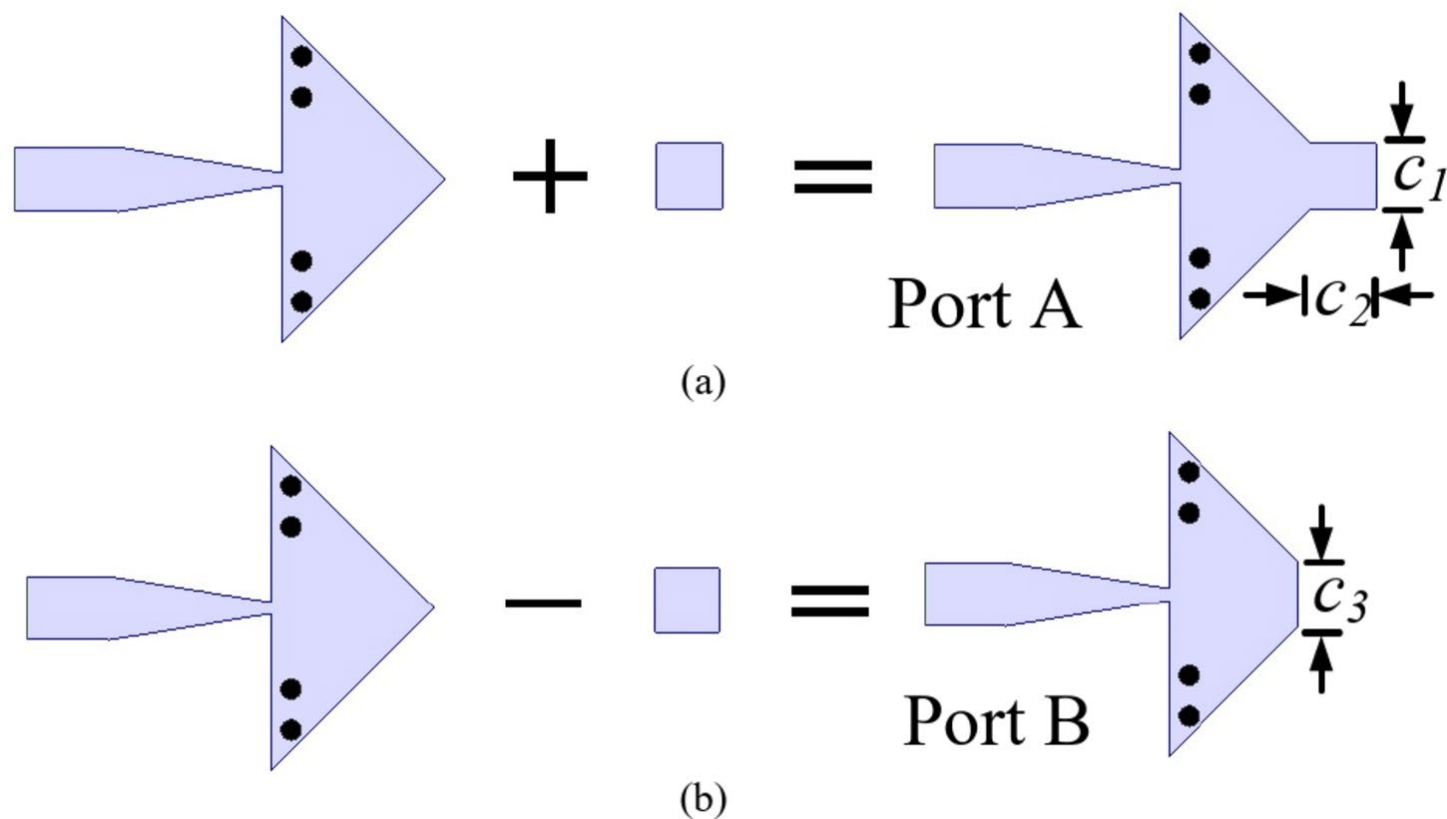
Modified QMSIW cavity resonator with feed: (**a**) for lower frequencies, (**b**) for higher frequencies.

Figure 2 shows design stages of QMSIW cavity resonator. Initially, a square SIW cavity of dimensions 4.1 mm × 4.1 mm is chosen. The diameter of the vias is 0.25 mm and their separation are 0.5 mm is chosen for the confinement of the electromagnetic fields within the cavity. The dominant mode for the square SIW cavity is TE_110_. With the usage of the magnetic wall concept along the diagonal symmetrical plane *AA*’, a half mode SIW (HMSIW) cavity resonator is obtained. The HMSIW resonator has symmetrical fields across *OB*’ plane. It also supports TE_110_ mode field configuration. By using the magnetic wall concept again, a QMSIW cavity resonator is obtained. The QMSIW cavity resonator is the basic building block for the design of proposed self-octaplexing antenna array. The QMSIW cavity resonator is fed through a microstrip feed line. In order to achieve self-octaplexing operations in n257, n258, n259 and n260 bands of 5G FR2 spectrum, each QMSIW cavity resonator must operate at distinct frequencies. To operate QMSIW cavity resonators at different frequencies, they are modified accordingly and are microstrip fed as shown in Fig. 3.

**Fig. 4:**
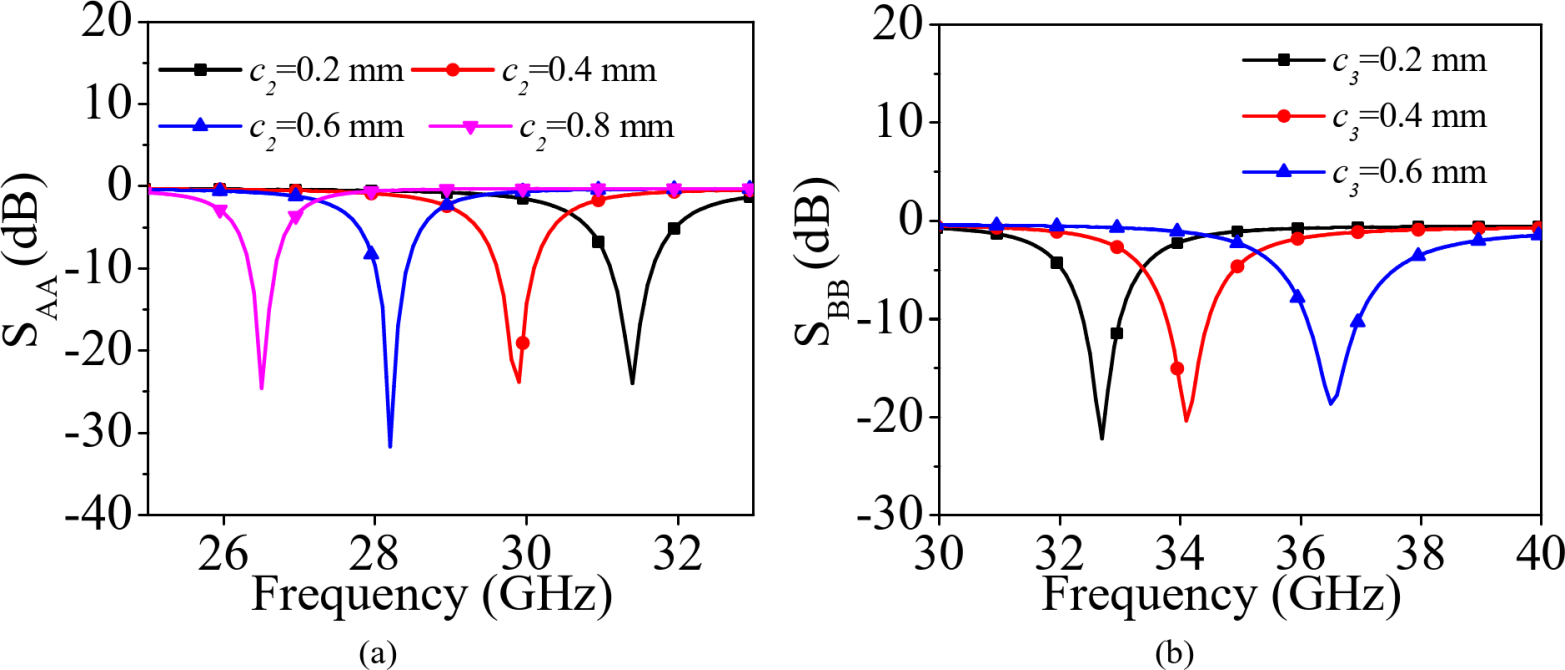
Parametric variation of (**a**) *c*_*2*_ and (**b**) *c*_*3*_.

**Fig. 5:**
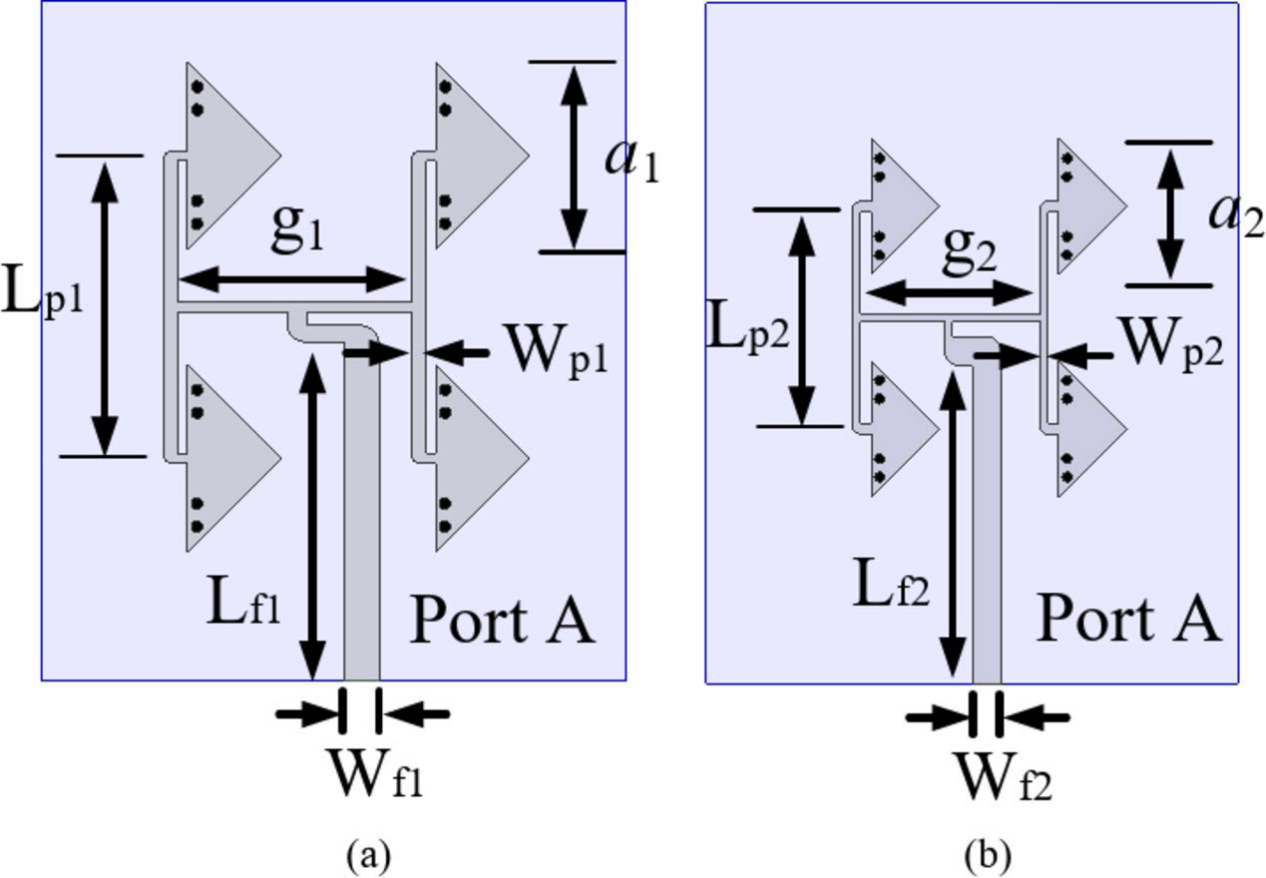
Unit element arrays: (**a**) UE1 for lower FR2 band, (**b**) UE2 for higher FR2 band (*a*_1_ = 4.1 mm, *a*_2_ = 3.49 mm, *g*_1_ = 5.06 mm, *g*_2_ = 4.62 mm, *Lp*_1_ = 6.59 mm, *Lp*_2_ = 5.71 mm, *Wp*_1_ = 0.32 mm, *Wp*_2_ = 0.21 mm, *Lf*_1_ = 8.34 mm, *Lf*_2_ = 7.86 mm, *Wf*_1_ = 0.78 mm, *Wf*_2_ = 0.74 mm).

**Fig. 6:**
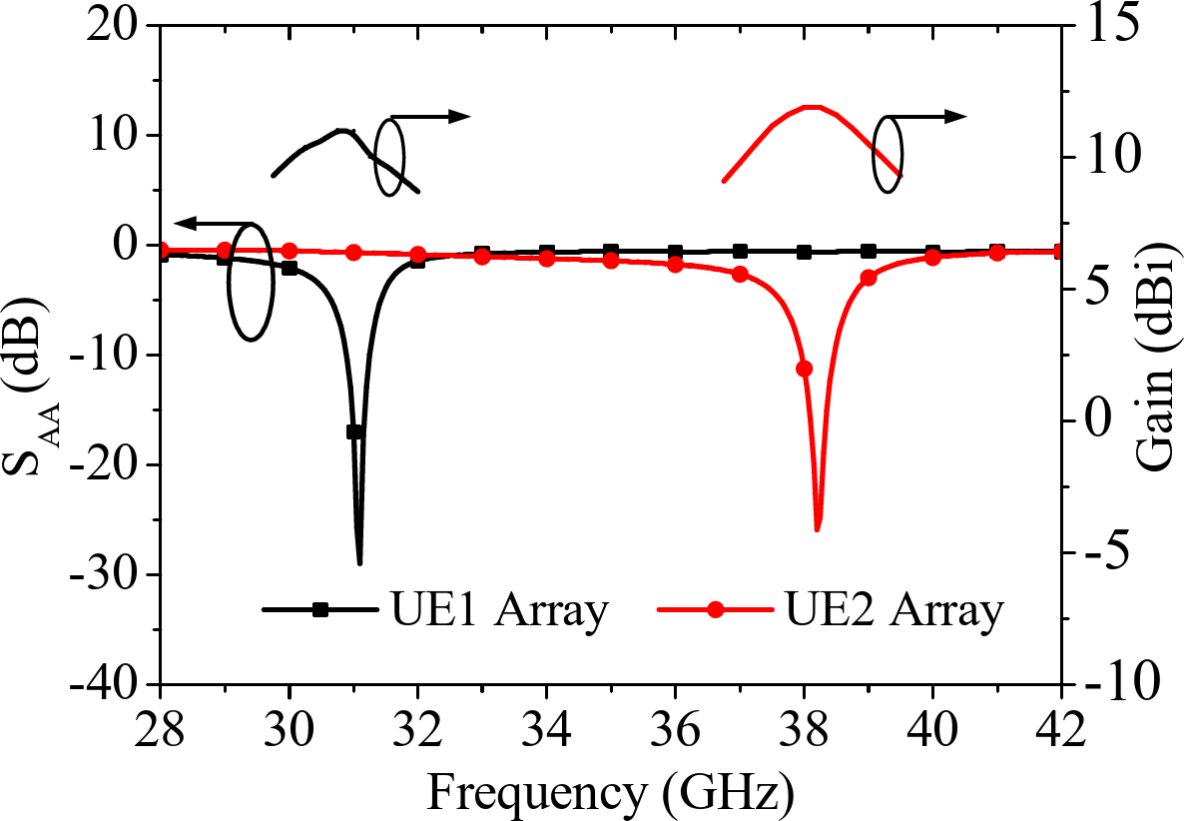
S-parameters for UE1 and UE2 along with their corresponding peak realized gains.

Figure 3(a) represents the modified QMSIW cavity resonator-based antenna element for the lower frequencies of 5G FR2 spectrum (26–32 GHz). Here, a rectangular strip of dimensions *c*_1_ × *c*_2_ is added on the QMSIW cavity resonator. The addition of this rectangular stub basically adds an additional capacitance that gradually decreases the resonant frequency of the QMSIW resonator. Figure 4(a) shows the *S*_*AA*_-parameters for the modified QMSIW cavity resonator for different values of *c*_2_. As the value of *c*_2_ increases, the resonant frequency moves towards the lower values of 5G FR2 spectrum. For the radiations at higher frequencies of 5G FR2 spectrum (36–42 GHz), the basic QMSIW cavity resonator is altered by removing a section of dimensions *c*_3_ as shown in Fig. 3(b). The *S*_*BB*_-parameters of the modified QMSIW cavity resonator for different values of *c*_3_ are plotted in Fig. 4(b). As the values of *c*_3_ increases, the effective area of QMSIW resonator decreases which shifts the operating frequencies toward the higher values of 5G FR2 spectrum. Thus, the resonant frequencies of the QMSIW cavity resonator can be independently tuned by the varying the design parameters *c*_2_ and *c*_3_.

**Fig. 7:**
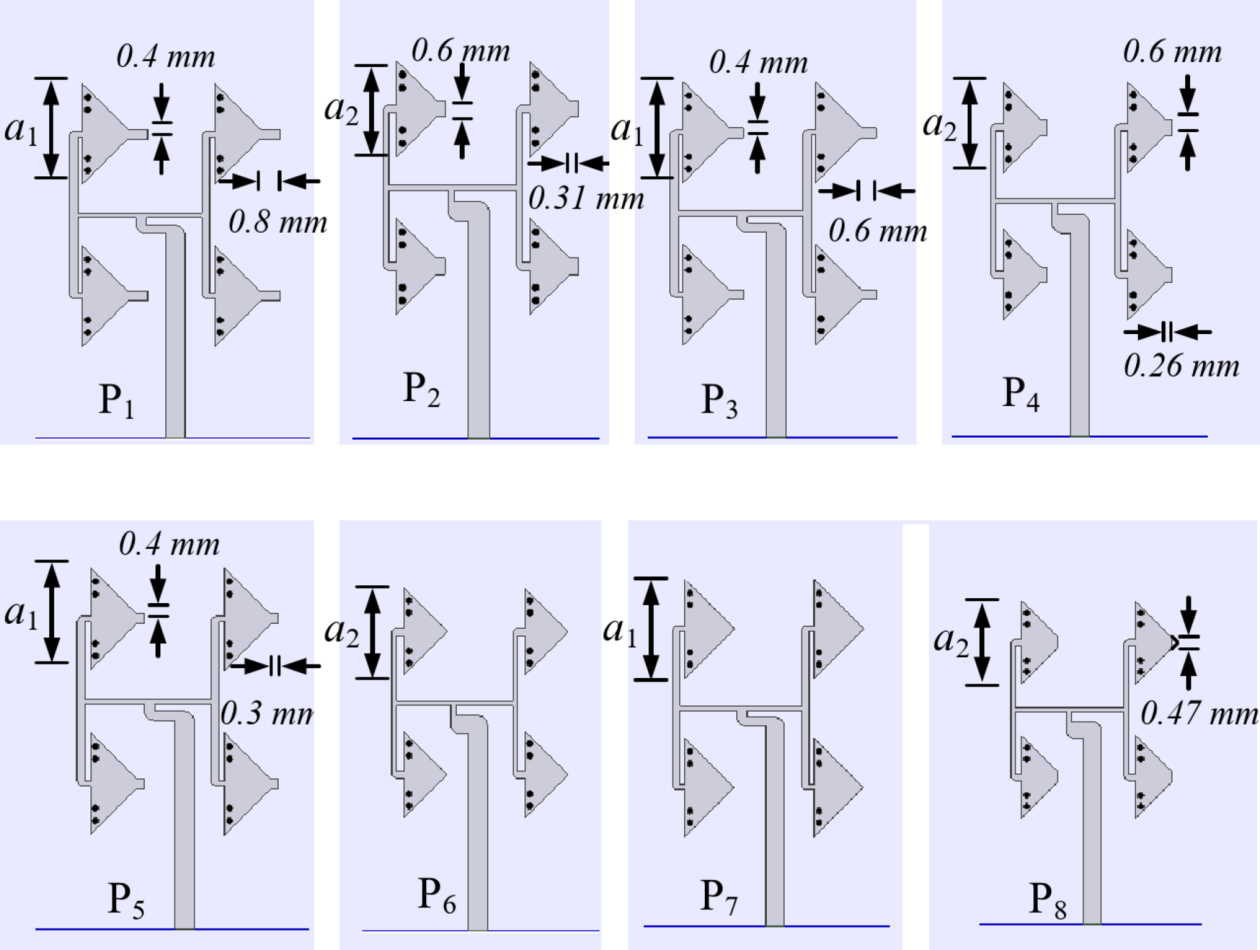
Unit Element arrays of the proposed self-multiplexing antenna array for each port (*a*_1_ = 4.1 mm, *a*_2_ = 3.49 mm).

## Unit element of self-octaplexing antenna array

In this sub-section, two four-element antenna arrays (one centered at 31 GHz and another centered at 38 GHz) are designed. The four-element antenna arrays are fed through a corporate feeding network. The corporate feeding network consists of two-stage T-junction power dividers. For good impedance matching, a *λ*/4 impedance transformer are added at each T-junction of the power dividers. The good impedance matching (|*S*_*ii*_| < −18 dB) is obtained at each input ports as well as equal and in-phase signals are obtained at the output ports of the feed network.

Further, the four QMSIW cavity resonators and the feed networks are combined together to create the four-element antenna array, that act as the unit element (UE) of the proposed self-octaplexing antenna array, as depicted in Fig. 5. In this work, two UEs, designated as UE1 (Fig. 5(a)) and UE2 (Fig. 5(b)) are designed for 31 GHz (lower 5G FR2 spectrum) and 38 GHz (upper 5G FR2 spectrum) frequencies, respectively. The main difference between these two UEs are the dimensions of the QMSIW cavity resonators and their feeding network. The dimensions of the UEs are provided in the caption of Fig. 5. The distance between the QMSIW cavity resonators of the unit element UE1 are 5.38 mm and 6.59 mm along the *x*-axis and *y*-axis, respectively. While the corresponding distances between the resonators of the unit element UE2 are 4.83 mm and 5.71 mm along the *x*-axis and *y*-axis, respectively. Figure 6 shows the S-parameters along with their corresponding gains of the unit elements of the proposed self-octaplexing antenna array. The UE1 radiates at 31 GHz, while UE2 radiates at 38 GHz with the respective gains of 11.1 dBi and 12 dBi.

**Fig. 8:**
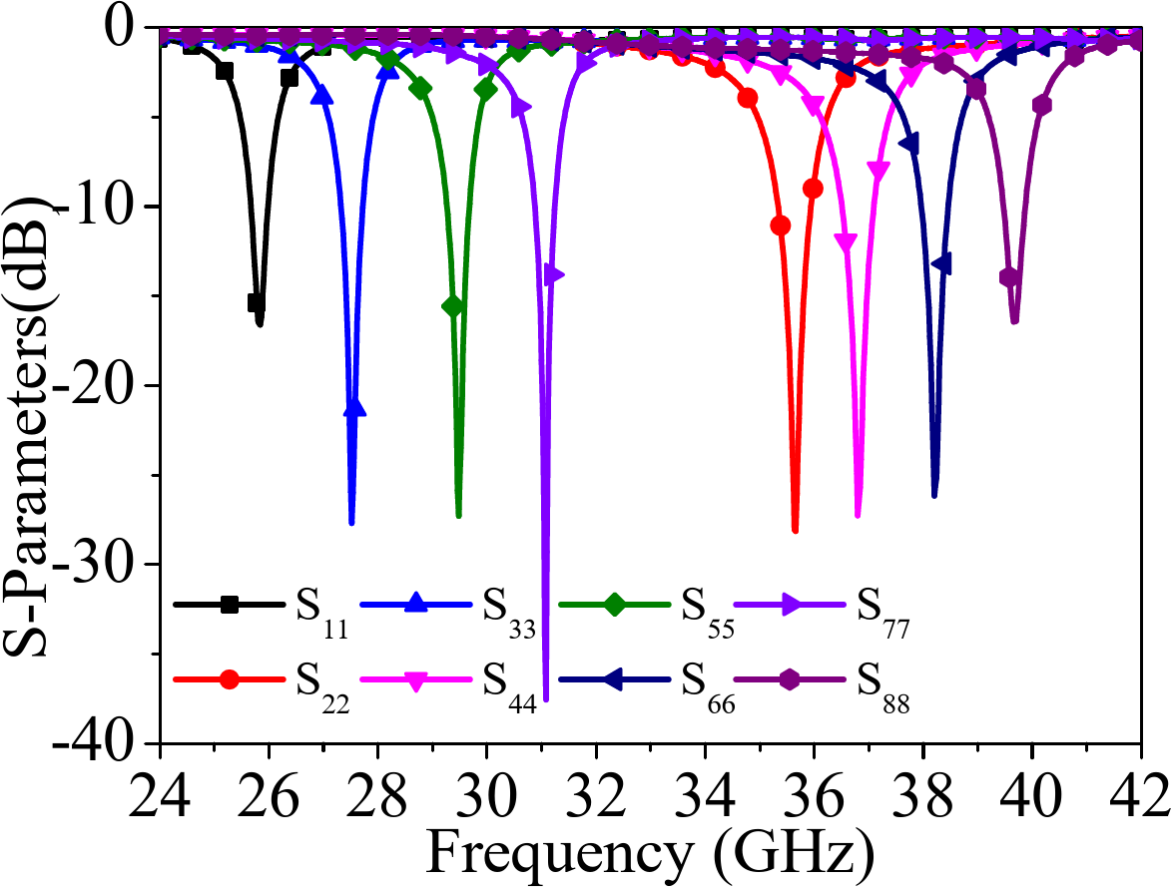
*S*_*ii*_-parameters of the proposed self-octaplexing antenna array.

**Fig. 9:**
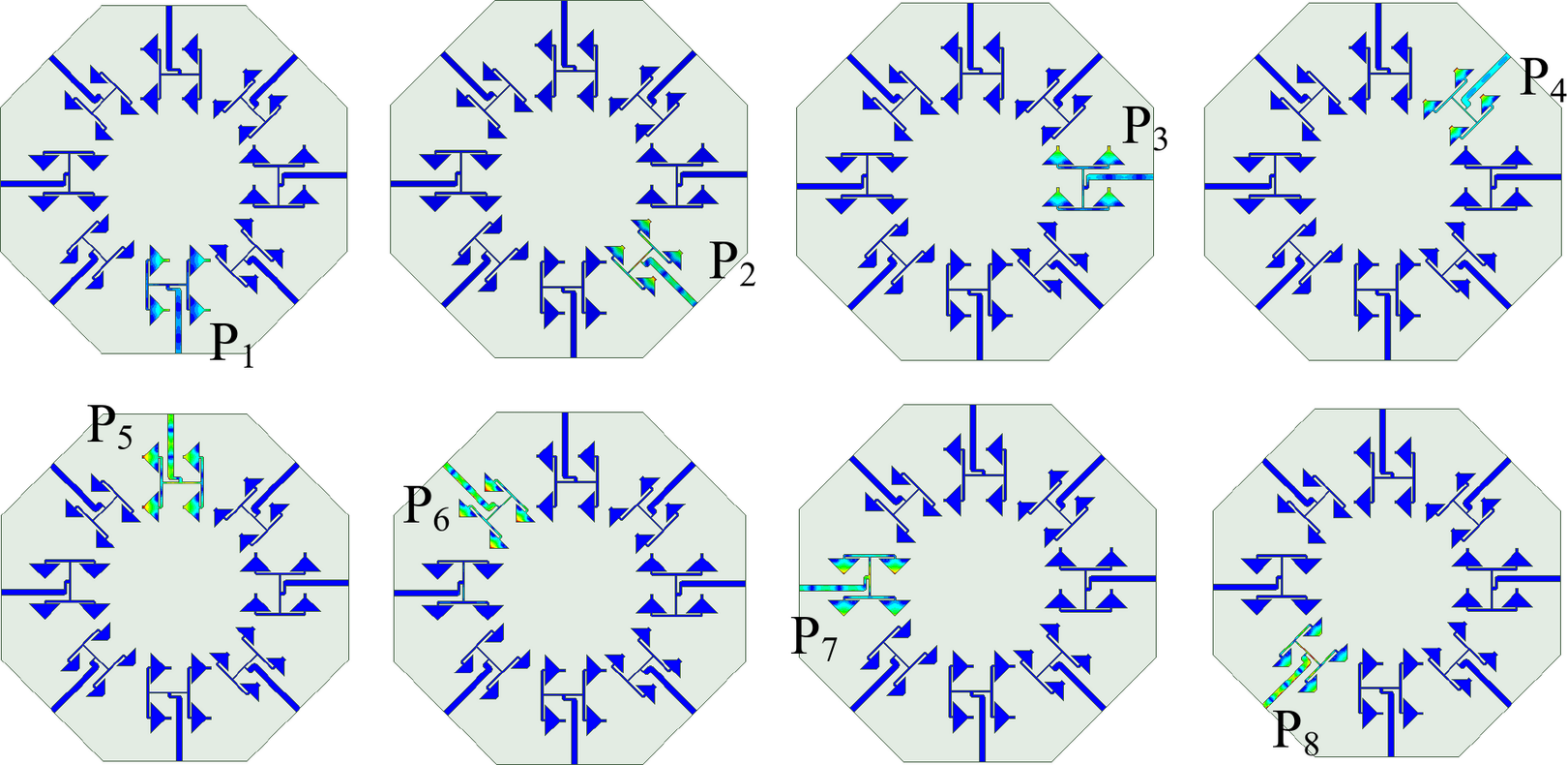
E-field distributions under different individual ports excitations at their respective operating frequencies.

## Proposed self-octaplexing antenna array

To build the proposed self-octaplexing antenna array, the unit element array UE1 is chosen as basic element for Port P_1_, Port P_3_, Port P_5_ and Port P_7_ (lower 5G FR2 spectrum), whereas unit element array UE2 is the basic element for Port P_2_, Port P_4_, Port P_6_ and Port P_8_ (higher 5G FR2 spectrum). The alternate placement of UE1 and UE2 ensures excellent inter-port isolations (> 33 dB) because their operating frequencies are far enough (e.g. Port P_1_ radiates at 25.8 GHz and Port P_2_ radiates at 35.6 GHz). The eight distinct frequencies of the self-octaplexing antenna arrays are obtained by choosing the different values of *c*_1_, *c*_2_ and *c*_3_. Figure 7 demonstrates the UEs for each port along with their dimensions. The distinct unit element arrays are placed across the sides of a regular octagon to obtain proposed self-octaplexing antenna array (Fig. 1) that facilitates electromagnetic radiations at eight distinct frequencies which lies in n257, n258, n259 and n260 bands of 5G FR2 spectrum.

**Fig. 10:**
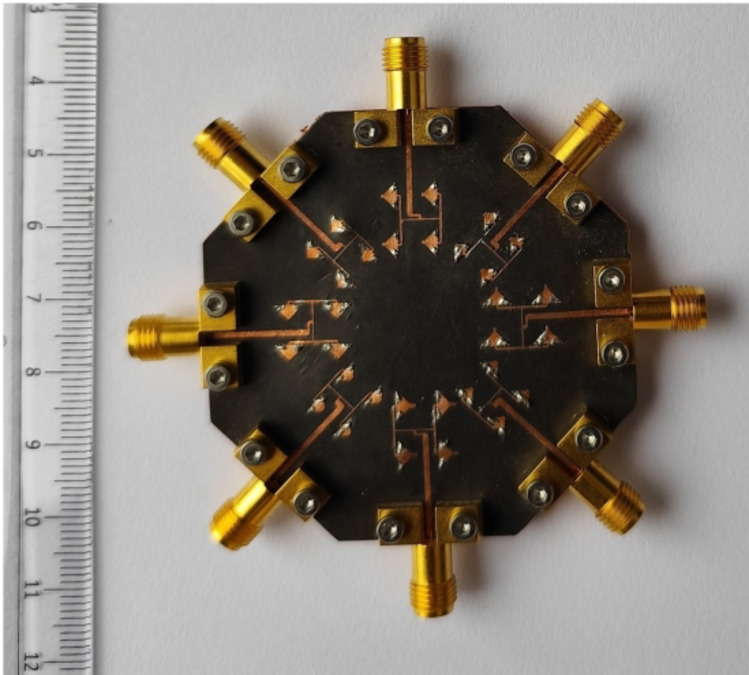
Fabricated prototype of the designed self-octaplexing antenna array.

Figure 8 shows the simulated S_*ii*_-parameters (*i* = 1, 2,…8) of the designed self-octaplexing antenna array as shown in Fig. 1. It radiates at 25.8 GHz, 27.5 GHz, 29.5 GHz, 31 GHz, 35.6 GHz, 36.8 GHz, 38.2 GHz, 39.7 GHz, when the ports P_1_, P_2_,…P_8_ are excited individually. The port-to-port isolations are better than 33 dB among all the ports of the antenna system. The attractive feature of the proposed self-octaplexing antenna array is that it doesn’t require any decoupling structure while maintaining the high inter-port isolations.

Figure 9 shows the electric-field distributions of the proposed self-octaplexing antenna array under different individual ports excitations at their corresponding operating frequencies. It can be observed from the figure that when one of the port (say P_1_) is excited, there is hardly any fields present at the other ports (P_2_, P_3_,…P_8_), that ensures high inter-port isolations. Moreover, it is also clear from the field distributions that these QMSIW antenna elements utilizes the dominant TE_110_ mode for radiations.

The design guidelines of the proposed self-octaplexing antenna array are as follows:

- Select two square SIW cavities, one for 31 GHz and another for 38 GHz. The dimensions of these SIW cavities are selected such that they support the dominant TE_110_ modes at 31 GHz and 38 GHz.
- Bifurcated twice the square SIW cavities by applying the magnetic wall concept along the symmetrical planes to obtain the miniaturized QMSIW cavities.
- Modify the QMSIW cavities for self-multiplexing operations either by adding or subtracting a rectangular patch from the vertices of the cavities to obtain eight distinct frequencies.
- Design two 2 × 2 T-junction power dividers, one at 31 GHz and another at 38 GHz. The power dividers will be utilized for feeding of UE1 and UE2.
- Place the eight distinct UE arrays across the edges of a regular octagon to obtain the proposed self-multiplexing antenna array that operates in eight distinct frequencies of 5G FR2 spectrum.

## Results and discussion

**Fig. 11:**
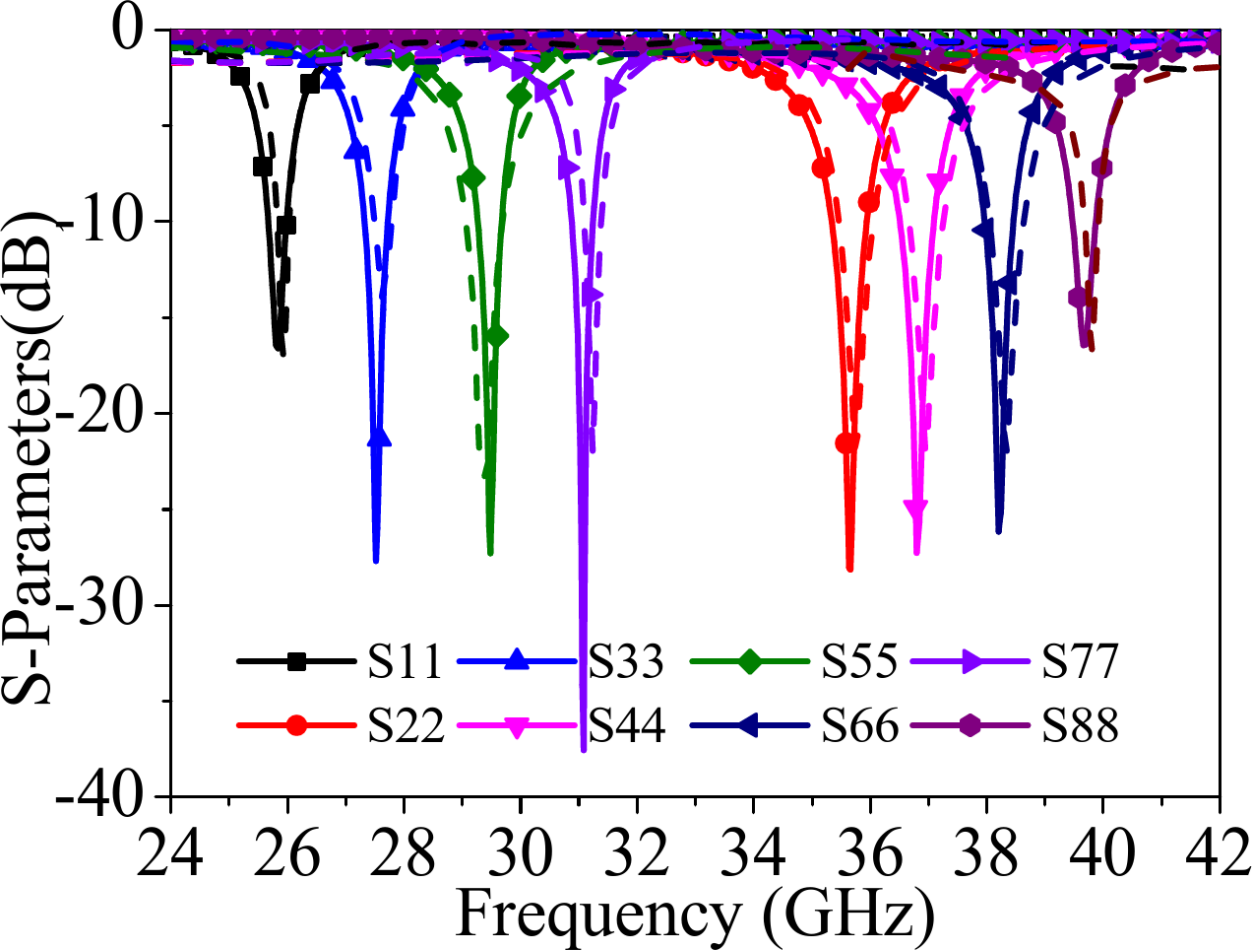
Simulated and measured *Sii* -parameters (*i* = 1, 2,…8) of the proposed self-octaplexing antenna array.

**Fig. 12:**
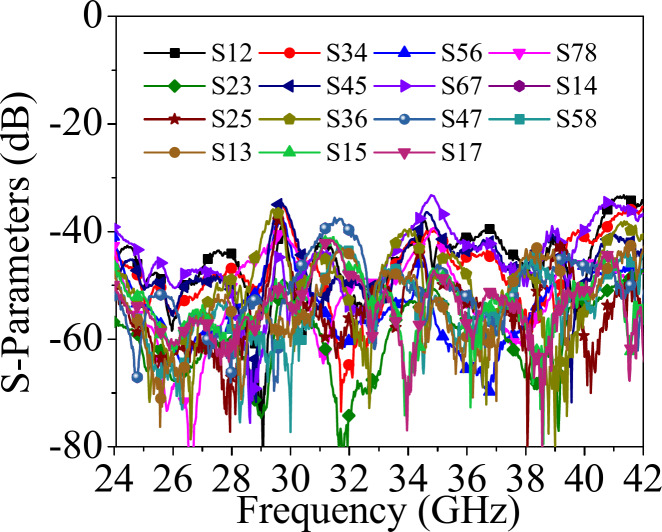
Measured *Sii*-parameters (*i* ≠ *j* , *i* , *j* = 1, 2,…8) of the proposed self-octaplexing antenna array.

Figure 10 shows the fabricated prototype of the designed self-octaplexing antenna array for 5G FR2 spectrum. It is fabricated on RT/duroid 5880 high frequency laminate with relative permittivity of 2.2, thickness of 0.254 mm and loss tangent of 0.0008. It is fabricated using standard PCB technique using LPKF Protomat machine. The vias are filled with copper wires of diameter 0.25 mm. Eight 02K243-40ME3 Rosenberger RF connectors are connected for the measurement purposes. The simulated and the measured S_*ii*_-parameters (*i* = 1, 2,…8) of the self-octaplexing antenna array are plotted in Fig. 11. The simulated (measured) operating frequencies of the self-octaplexing antenna array are 25.8 GHz (25.94 GHz), 27.5 GHz (27.62 GHz), 29.5 GHz (29.42 GHz), 31 GHz (31.2 GHz), 35.6 GHz (35.76 GHz), 36.8 GHz (36.96 GHz), 38.2 GHz (38.32 GHz) and 39.7 GHz (39.84 GHz) that lies in n257, n258, n259 and n260 of 5G spectrum. The measured inter-port isolation values are shown in Fig. 12. The frequencies of Port P_1_, Port P_3_, Port P_5_ and Port P_7_ are closer to each other, still high isolation (> 33 dB) among them is observed. High isolations can be attributed towards the orthogonal placement of the ports, namely Port P_1_ is orthogonal to Port P_3_ as well as Port P_7_. The distance between Port P_1_ and Port P_5_ is approximately 2.6*λ*_0_, where *λ*_0_ is the wavelength at 28 GHz. The large spacing between these port pairs is responsible for high isolation (> 33 dB) between them. The isolation among all the port pairs is better than 33 dB, that guarantees the efficient self-multiplexing operation of the proposed antenna.

**Fig. 13:**
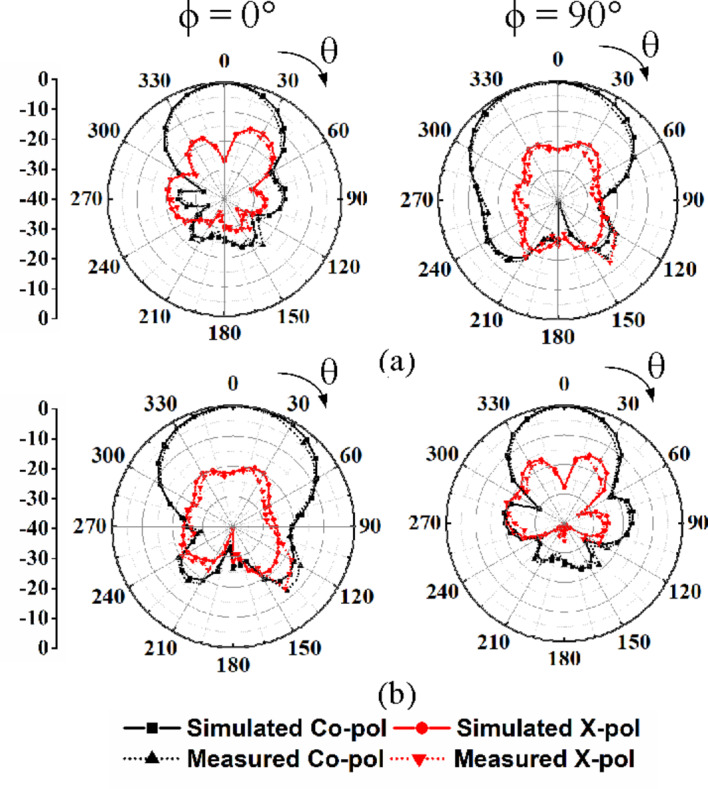
Far-field radiation patterns of the proposed self-octaplexing antenna array: (a) under Port P_1_ excitation, (b) under Port P_3_ excitation.

**Fig. 14:**
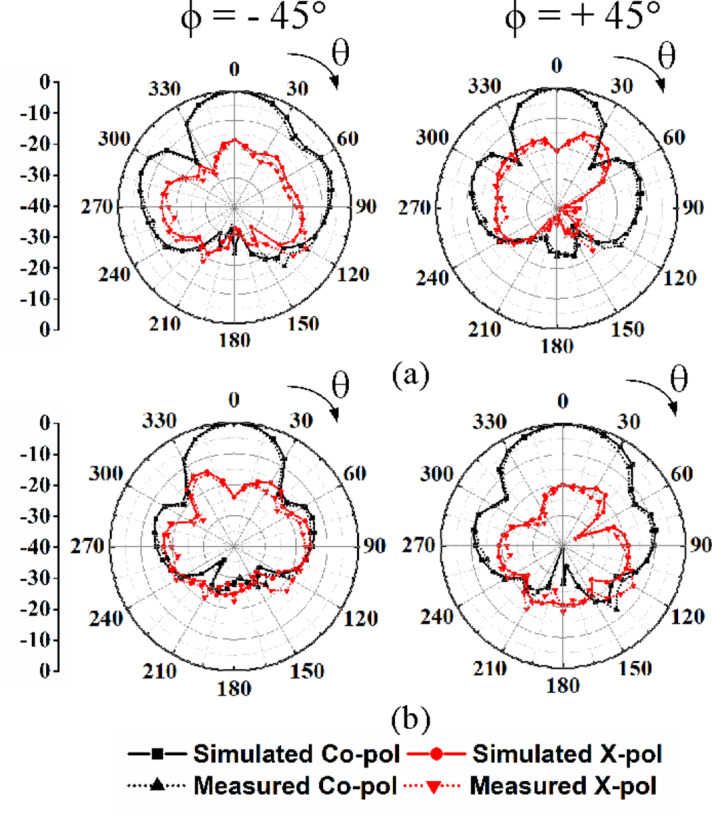
Far-field radiation patterns of the proposed self-octaplexing antenna array: (a) under Port P_2_ excitation, (b) under Port P_4_ excitation.

**Fig. 15:**
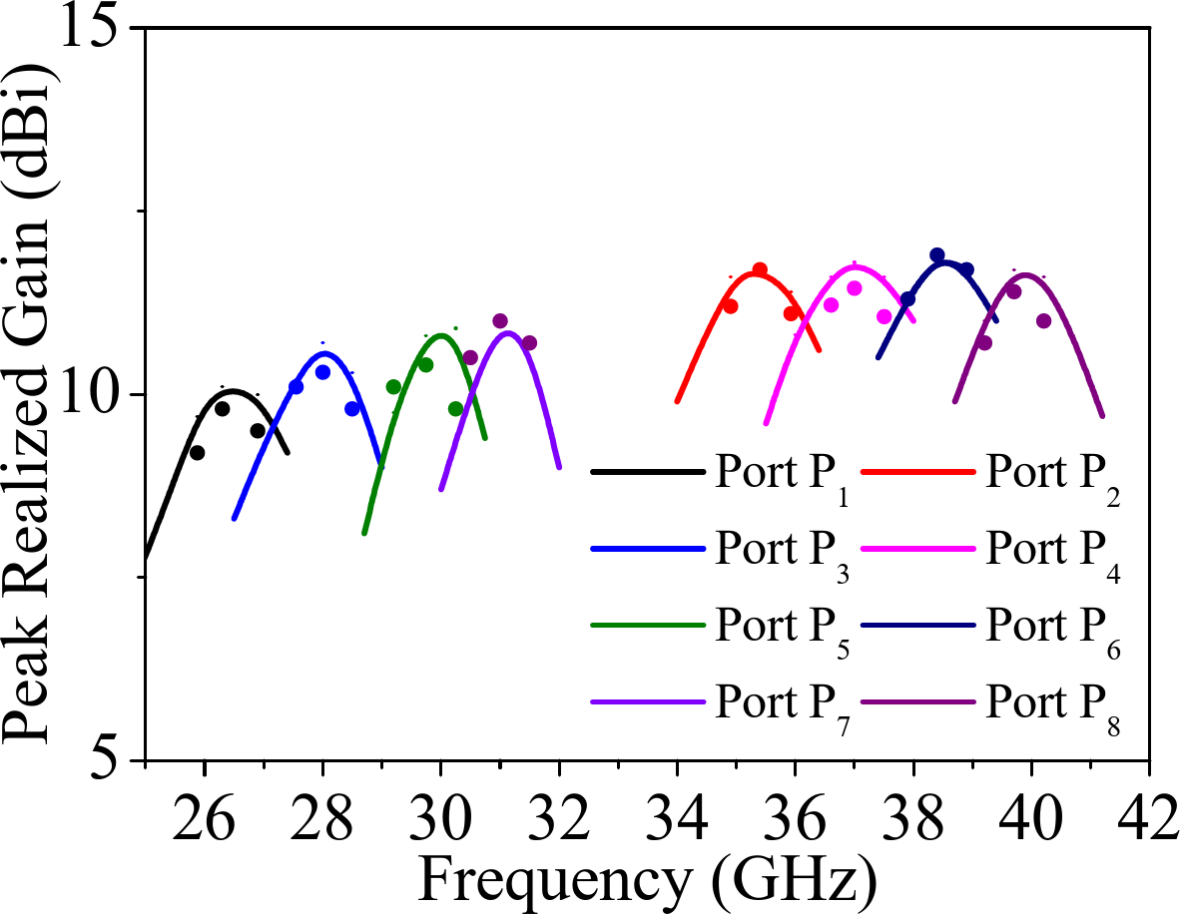
Peak realized gains of the proposed self-octaplexing antenna array (solid: simulated, dotted: measured).

**Fig. 16:**
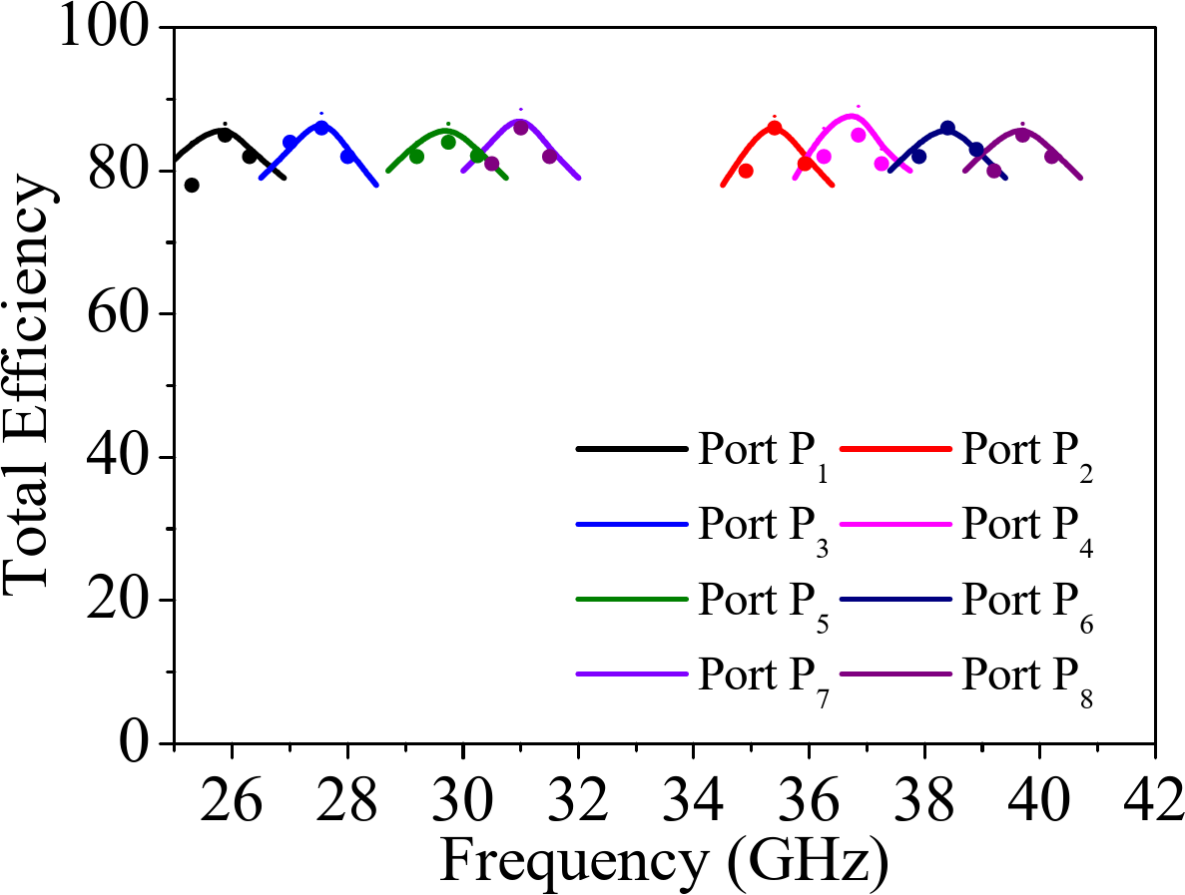
Simulated and measured efficiency of the proposed self-octaplexing antenna array (solid: simulated, dotted: measured).

**Table 1:** Comparison table.

Ref.	No. of Bands	Frequency (GHz)	Isolation (dB)	Gain (dBi)	mm-Wave Operation
^19^	5	2.29, 2.98, 3.65, 4.37, 5.08	29	3.59, 4.55, 3.91, 5.7, 4.92	No
^20^	6	5.33, 5.76, 6.31, 6.86, 7.34, 7.8	24	4.5, 4.94, 4.9, 5.12, 6.12, 6.6	No
^21^	6	2.29, 2.96, 4.30, 5.0, 5.61, 6.18	27	3.73, 4.35, 5.57, 5.46, 5, 4.73	No
^23^	6	4, 5.8, 6.6, 7.8, 9.8, 10.62	27	4.9, 5.11, 5.4, 5.43, 5.32, 5.3	No
^24^	8	3.54, 3.81, 4.12, 4.5, 4.85, 5.22, 5.5, 5.87	30	4.07, 4.3, 4.72, 5.11, 4.43, 4.51, 4.97, 4.47	No
^25^	8	5.15, 5.67, 6.18, 6.6, 7.18, 7.85, 8.25, 8.85	20	3.9, 3.2, 4.05, 4.14, 3.8, 3.37, 3.55, 3.28	No
^26^	8	3.35, 3.58, 3.73, 3.91, 4.09, 4.32, 4.61, 4.96	23	4.53, 4.62, 4.67, 4.74, 3.83, 3.89, 3.42, 3.55	No
^30^	4	5.8, 7.4, 28, 38	26	4.1, 5.2, 6.1, 8.3	Partial
^31^	4	4.8, 5.4, 28, 30	20	5.4, 5.2, 8, 8.7	Partial
^32^	4	24.25, 26.5, 27.5, 29.5	20	4.45, 4.3, 3.7, 3.84	Yes
Prop.	8	25.8, 27.5, 29.5, 31, 35.6, 36.8, 38.2, 39.7	29	9.7, 10.7, 10.9, 11.0, 11.4, 11.7, 11.9, 12.2	Yes

The normalized far field radiation patterns of the proposed antenna systems for only first four ports at their corresponding self-multiplexing frequencies are shown. Figure 13 shows the simulated and measured radiation patterns under Port P_1_ and P_3_ excitations in *ϕ* = 0° and *ϕ* = 90° planes at the respective frequencies of 25.8 GHz and 27.5 GHz, whereas Fig. 14 demonstrates the radiation patterns under Port P_2_ and P_4_ excitations in *ϕ* = −45° and *ϕ* = +45° planes at the corresponding frequencies of 35.6 GHz and 36.8 GHz. The radiation patterns are broadside in nature with high front-to-back ratio (FTBR). The cross-polarization level is less than −20 dB with respect to co-polarization level at the broadside direction. The peak realized gains of the proposed self-octaplexing antenna array are plotted in Fig. 15. It can be observed that the designed antenna array exhibits peak realized gains of 9.7, 10.7, 10.9, 11.0, 11.4, 11.7, 11.9 and12.2 dBi when ports P_1_, P_2_, P_3_, P_4_, P_5_, P_6_, P_7_ and P_8_ are excited at their respective operating frequencies. The simulated and measured total efficiencies of the proposed self-octaplexing antenna array are greater than 85%, as shown in Fig. 16.

Table 1demonstrates the merits of proposed self-octaplexing antenna array with respect to other reported self-multiplexing antennas for microwave and millimeter wave bands. As compared to references^19–23^, the proposed antenna offers self-multiplexing operations in larger number of frequency bands. The self-multiplexing antennas presented in references^24–26^operate in eight distinct microwave frequencies, while the proposed antenna have self-multiplexing characteristics in millimeter wave frequencies along with higher operating bandwidth. The self-quadruplexing antennas in references^30,31^are partially operating in millimeter wave frequencies, while the self-quadruplexing antenna in reference^32^ operates fully at four distinct millimeter wave frequency bands. The above comparison shows that the proposed antenna performs self-multiplexing operations in eight distinct millimeter wave frequencies with higher bandwidth and excellent inter-port isolation, as well as high gains, making it a potential candidate for communication systems operating in the 5G FR2 spectrum. Moreover, to the best of the authors’ knowledge, this is the first instance where the self-octaplexing antenna array for millimeter wave frequencies is designed and developed.

The self-octaplexing antenna array is designed to operate at in the 5G FR2 n257, n258, n259 and n260 spectrum bands. The antenna has eight distinct operating frequencies: 25.8 GHz, 27.5 GHz, 29.5 GHz, 31 GHz, 35.6 GHz, 36.8 GHz, 38.2 GHz and 39.7 GHz. As specified by 3GPP33, millimeter wave frequencies (24.25 GHz to 52.6 GHz) are ideal for internet of things (IoT) applications that require high data rates and low latency. Hence, the proposed high gain self-octaplexing antenna array could be an attractive choice for ensuring high-speed data transmission in 5G FR2 communication scenarios. It can also be redesigned for other millimeter wave 5G FR2 frequencies (n257, n258, n259 and n260 bands) by changing the dimensions of the QMSIW cavity resonators.

## Conclusion

In this article, a self-octaplexing antenna array for 5G FR2 spectrum is designed and developed. The unit element array comprises of four QMSIW cavity resonators which are fed through a corporate feed network. Each unit element array is placed across the edge of a regular octagon. The designed self-octaplexing antenna array radiates at eight distinct frequencies of 25.8 GHz, 27.5 GHz, 29.5 GHz, 31 GHz, 35.6 GHz, 36.8 GHz, 38.2 GHz, 39.7 GHz with respective gains of 9.7, 10.7, 10.9, 11.0, 11.4, 11.7, 11.9, 12.2 dBi. The high inter-port isolations (> 33 dB), high gains and high FTBRs of the designed self-octaplexing antenna array makes it a potential candidate for wireless communication systems that operates in 5G FR2 spectrum.

## Data Availability

The datasets used and/or analysed during the current study available from the corresponding author on reasonable request.
